# Improving the Clinical Outcome in Stroke Patients Receiving Thrombolytic or Endovascular Treatment in Korea: from the SECRET Study

**DOI:** 10.3390/jcm9030717

**Published:** 2020-03-06

**Authors:** Young Dae Kim, Ji Hoe Heo, Joonsang Yoo, Hyungjong Park, Byung Moon Kim, Oh Young Bang, Hyeon Chang Kim, Euna Han, Dong Joon Kim, JoonNyung Heo, Minyoung Kim, Jin Kyo Choi, Kyung-Yul Lee, Hye Sun Lee, Dong Hoon Shin, Hye-Yeon Choi, Sung-Il Sohn, Jeong-Ho Hong, Jang-Hyun Baek, Gyu Sik Kim, Woo-Keun Seo, Jong-Won Chung, Seo Hyun Kim, Tae-Jin Song, Sang Won Han, Joong Hyun Park, Jinkwon Kim, Yo Han Jung, Han-Jin Cho, Seong Hwan Ahn, Sung Ik Lee, Kwon-Duk Seo, Hyo Suk Nam

**Affiliations:** 1 Department of Neurology, Yonsei University College of Medicine, Seoul 03722, Korea; neuro05@yuhs.ac (Y.D.K.); jhheo@yuhs.ac (J.H.H.); JSYOO@yuhs.ac (J.Y.); hjpark209042@gmail.com (H.P.); jnheo@jnheo.com (J.H.); bestmykim@gmail.com (M.K.); JKSNAIL85@yuhs.ac (J.K.C.); 2 Department of Neurology, Brain Research Institute, Keimyung University School of Medicine, Daegu 41931, Korea; sungil.sohn@gmail.com (S.-I.S.); neurohong79@gmail.com (J.-H.H.); 3 Department of Radiology, Yonsei University College of Medicine, Seoul 03722, Korea; BMOON21@yuhs.ac (B.M.K.); DJKIMMD@yuhs.ac (D.J.K.); 4 Department of Neurology, Samsung Medical Center, Sungkyunkwan University School of Medicine, Seoul 06351, Korea; ohyoung.bang@samsung.com (O.Y.B.); mcastenosis@gmail.com (W.-K.S.); neurocjw@gmail.com (J.-W.C.); 5 Department of Preventive Medicine, Yonsei University College of Medicine, Seoul 03722, Korea; hckim@yuhs.ac; 6 College of Pharmacy, Yonsei Institute for Pharmaceutical Research, Yonsei University, Incheon 21983, Korea; eunahan@yonsei.ac.kr; 7 Department of Neurology, Gangnam Severance Hospital, Severance Institute for Vascular and Metabolic Research, Yonsei University College of Medicine, Seoul 06273, Korea; KYLEE@yuhs.ac (K.-Y.L.); antithrombus@gmail.com (J.K.); 8 Department of Research Affairs, Biostatistics Collaboration Unit, Yonsei University College of Medicine, Seoul 06273, Korea; HSLEE1@yuhs.ac; 9 Department of Neurology, Gachon University Gil Medical Center, Incheon 21565, Korea; sphincter@naver.com; 10 Department of Neurology, Kyung Hee University Hospital at Gangdong, Kyung Hee University School of Medicine, Seoul 05278, Korea; hyechoi@gmail.com; 11 Department of Neurology, National Medical Center, Seoul 04564, Korea; janghyun.baek@gmail.com; 12 Department of Neurology, Kangbuk Samsung Hospital, Sungkyunkwan University School of Medicine, Seoul 03181, Korea; 13 Department of Neurology, National Health Insurance Service Ilsan Hospital, Ilsan 10444, Korea; gskim@nhimc.or.kr (G.S.K.); seobin7@naver.com (K.-D.S.); 14 Department of Neurology, Yonsei University Wonju College of Medicine, Wonju 26426, Korea; s-hkim@yonsei.ac.kr; 15 Department of Neurology, Seoul Hospital, Ewha Womans University College of Medicine, Seoul 07804, Korea; knstar@hanmail.net; 16 Department of Neurology, Sanggye Paik Hospital, Inje University College of Medicine, Seoul 01757, Korea; sah1puyo@gmail.com (S.W.H.); truelove1@hanmail.net (J.H.P.); 17 Department of Neurology, CHA Bundang Medical Center, CHA University, Seongnam 13496, Korea; 18 Department of Neurology, Changwon Fatima Hospital, Changwon 51394, Korea; eyasyohan@gmail.com; 19 Department of Neurology, Pusan National University School of Medicine, Busan 49241, Korea; chohj75@gmail.com; 20 Department of Neurology, Chosun University School of Medicine, Gwangju 61453, Korea; shahn@Chosun.ac.kr; 21 Department of Neurology, Sanbon Hospital, Wonkwang University School of Medicine, Sanbon 15865, Korea; neurologist@hanmail.net

**Keywords:** reperfusion, therapy, ischemic stroke, outcome

## Abstract

We investigated whether there was an annual change in outcomes in patients who received the thrombolytic therapy or endovascular treatment (EVT) in Korea. This analysis was performed using data from a nationwide multicenter registry for exploring the selection criteria of patients who would benefit from reperfusion therapies in Korea. We compared the annual changes in the modified Rankin scale (mRS) at discharge and after 90 days and the achievement of successful recanalization from 2012 to 2017. We also investigated the determinants of favorable functional outcomes. Among 1230 included patients, the improvement of functional outcome at discharge after reperfusion therapy was noted as the calendar year increased (*p* < 0.001). The proportion of patients who were discharged to home significantly increased (from 45.6% in 2012 to 58.5% in 2017) (*p* < 0.001). The successful recanalization rate increased over time from 78.6% in 2012 to 85.1% in 2017 (*p* = 0.006). Time from door to initiation of reperfusion therapy decreased over the years (*p* < 0.05). These secular trends of improvements were also observed in 1203 patients with available mRS data at 90 days (*p* < 0.05). Functional outcome was associated with the calendar year, age, initial stroke severity, diabetes, preadmission disability, intervals from door to reperfusion therapy, and achievement of successful recanalization. This study demonstrated the secular trends of improvement in functional outcome and successful recanalization rate in patients who received reperfusion therapy in Korea.

## 1. Introduction

Stroke is one of the diseases with the highest burden worldwide. Although the age-standardized risk of stroke or case fatality has been improving, there is still an increase in the absolute number of stroke or stroke-related death [1]. The Global Burden of Disease Study demonstrated that the burden of cerebrovascular disease increased over several decades and ranked second in the highest burden of diseases in 2015 [2]. 

Intravenous tissue plasminogen activator (IV t-PA) therapy and endovascular treatment (EVT) are established treatments for eligible patients with acute ischemic stroke [3,4]. Although these modalities can lead to successful recanalization, which is a strong determinant of a good outcome [5], many patients who received reperfusion therapy did not achieve a favorable outcome [6]. Over the past decades, there has been an improvement in the stroke care program, imaging techniques, treatment devices, and experience in EVT. As a result, overall outcome of reperfusion therapy for acute ischemic stroke could be improved at a national level [7]. In Korea, there have been improvements in the care system for acute stroke patients, including easy and rapid accessibility to medical services, establishment of stroke units or centers, and acute stroke codes for reperfusion therapy [8,9,10]. Considering these secular trends in the stroke care system, clinical and radiologic outcomes after reperfusion therapy might have changed in Korea.

We investigated whether there was an annual change in outcomes in patients who received IV t-PA therapy or EVT in Korea. We also determined which factors had played a role in these changes using the nationwide thrombolytic and EVT registry. 

## 2. Materials and Methods

### 2.1. Patients Inclusion

The study population was derived from the Selection Criteria in Endovascular Thrombectomy and thrombolytic therapy (SECRET) registry (Clinicaltrials.gov NCT02964052, https://clinicaltrials.gov/ct2/show/NCT02964052?term=NCT02964052&rank=1). The SECRET registry is a nationwide multicenter registry for exploring the selection criteria of patients who would benefit from reperfusion therapies. The SECRET registry was started on May 2016. This registry consisted of four parts: (1) clinical information, (2) information on reperfusion therapy, (3) comorbidities, and (4) imaging data. 

The clinical information section includes the demographics, vascular risk factors, previous medication status, laboratory findings, and neurologic status or premorbidity before stroke. In the reperfusion therapy section, the information on time parameters, angiographic findings before and after treatment, devices used during the procedure, periprocedural complications, and concomitant thrombolytic agents used was collected. The modified Rankin scale (mRS) at discharge and after 90 days, along with mortality within 6 months, was determined in each patient during the follow-up. If a patient died, we also assessed the cause of death. 

For the comorbidities section, we determined the presence of the component of the Charlson comorbidity index (CCI) for each patient. In the stroke population, we used a modified version of the CCI, which consisted of 19 diseases, including myocardial infarction, congestive heart failure, peripheral vascular disease, previous stroke, atrial fibrillation, dementia, depression, chronic pulmonary disease, ulcer disease, mild liver disease, moderate or severe renal disease, connective tissue disease or rheumatic disease, anemia, diabetes, acquired immune deficiency syndrome, cancer, leukemia, lymphoma, and metastatic cancer [11].

The imaging data section included the occlusion site, infarction core, collateral status, and thrombus characteristics on thin-section computed tomography (CT). The imaging findings were ascertained by the imaging adjudication committee (6 stroke neurologists and 4 neuroradiologists). The audit was conducted every two weeks, and the data management center verified the completeness and accuracy of the data. For this study, we used the demographics, vascular risk factors, underlying vascular diseases, time parameters, occlusion site, angiographic findings before and after treatment, and functional outcome variables.

This registry included 1026 patients who had been registered retrospectively from 15 hospitals between January 2012 and December 2015 and 333 patients who had been registered prospectively from 13 hospitals between November 2016 and December 2017. For prospectively-enrolled patients, written informed consent was obtained from patients or the next of kin. This registry was approved by the institutional review board in each participating hospital.

### 2.2. Reperfusion Therapy

IV t-PA and EVT was used in patients who met the criteria based on current guidelines. IV t-PA (Actilyse; Boehringer-Ingelheim, Ingelheim, Germany) was used in patients who had a stroke within 4.5 h from symptom onset and met the criteria based on current guidelines with a standard dose (0.9 mg/kg) [12,13]. If patients had large vessel occlusion on initial angiographic studies and could be treated within 8 h from symptom onset, EVT was considered. The EVT was performed primarily using mechanical devices rather than chemical agents. Among the mechanical devices, Solitaire stent retriever (Medtronic Neurovascular, Iirvine, CA, USA), Trevo retriever (Stryker Neurovascular, Fremont, CA, USA), or Penumbra reperfusion catheter (Penumbra, Alameda, CA, USA) was available in Korea and used based on target vessel site, tortuosity, or neurointerventionalist’s preference. Intra-arterial thrombolysis with urokinase (Green Cross, Seoul, Korea) or glycoprotein IIb/IIIa antagonists was used as an adjuvant therapy in certain cases including those with re-occlusion or distal embolization. 

If the onset of symptom was unclear, EVT was performed based on imaging findings and physician’s discretion. Brain magnetic resonance imaging and magnetic resonance angiography were performed 24 h after reperfusion therapy. When brain MRI could not be performed, brain CT and/or CT angiography was performed. During hospitalization, each patient was treated on the basis of current stroke guidelines [14,15].

### 2.3. Outcome Measures

In this study, functional outcome was assessed with mRS at discharge and after 90 days. Favorable functional was defined as having mRS score of 0–2 and excellent functional outcomes was defined as mRS score of 0–1. In terms of radiologic outcomes, successful recanalization was determined using digital subtraction angiography (DSA), CT angiography (CTA), or magnetic resonance angiography (MRA). In this study, successful recanalization was defined as thrombolysis in cerebral infarction grade of 2b or 3 on final DSA among patients with EVT [16]. In patients who received IV t-PA only, successful recanalization was defined as arterial occlusive lesion (AOL) scoring of 3 on CTA or MRA performed within 24 h. Symptomatic intracerebral hemorrhage was defined as having any type of hemorrhage causing neurologic deterioration with National Institutes of Health Stroke Scale (NIHSS) score ≥4 or leading to death or surgery within 7 days of stroke onset based on the criteria in the European Cooperative Acute Stroke Study (ECASS) III trial [12]. 

### 2.4. Statistical Analysis

When we compared the baseline characteristics according to the calendar year, Student’s independent *t*-test was used to compare age, time interval, and laboratory findings, and Pearson’s χ^2^ test or Fisher’s exact test was used in the analysis of categorical data. Wilcoxon rank sum test was used to compare baseline NIHSS scores. Because the number of patients who received reperfusion therapy in December 2016 and registered in the SECRET registry was small, these patients were merged into the patient group treated in 2017 for this analysis. When we investigated the trends of outcomes by year, linear-by-linear or Jonckheere-Terpstra test was used for the analysis. To determine the independent predictors of outcomes, logistic regression or ordinal regression analysis was used. A multivariable analysis was performed using all variables with a *p*-value < 0.1 in the univariable analysis. All *p*-values were two-sided, and a *p*-value < 0.05 was considered statistically significant. All statistical analysis was performed using Windows SPSS package (version 23.0, IBM Corp., Armonk, NY, USA) and R version 3.2.1 (R Foundation for Statistical Computing, Vienna, Austria, http://www.R-project.org).

## 3. Results

### 3.1. Baseline Characteristics

**Figure 1:**
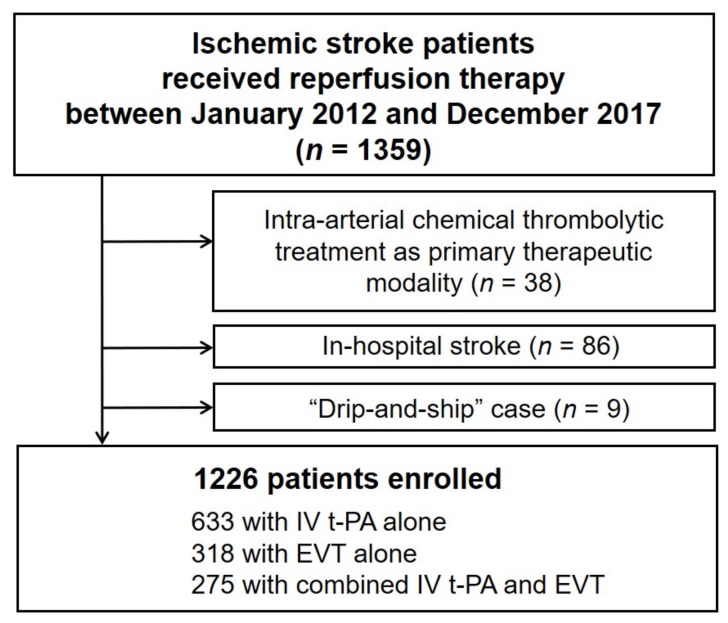
Flow diagram for the selection of patients in this study. IV, intravenous; t-PA, tissue plasminogen activator; EVT, endovascular.

Between January 2012 and December 2017, a total of 1359 patients who received reperfusion therapy with either IV t-PA therapy or EVT were registered. First, we excluded 38 patients who received intra-arterial chemical thrombolytic treatment as primary therapeutic modality. Then, we also excluded 86 patients who had in-hospital ischemic stroke, and nine patients who were transferred to the study hospital from other local hospitals (“drip-and-ship” case) because there were insufficient data on time parameters such as the intervals from stroke onset to first hospital arrival, CT, or IV t-PA. Finally, 1226 patients were included in this analysis (Figure 1).

Baseline characteristics of patients are presented in Table 1. The mean age was 68.9 ± 11.6 years, and 724 patients (58.9%) were male. The most common risk factor was hypertension (70.9%), followed by atrial fibrillation or atrial flutter (48.3%). The median NIHSS score was 12 (interquartile range [IQR], 7–17). Six-hundred and thirty-three (51.6%) patients received IV t-PA treatment alone, 318 (25.9%) patients received EVT alone, and the remaining 275 (22.4%) received combined IV t-PA and EVT. The number of patients who received IV t-PA and registered in SECRET registry was larger than those registered after EVT with/without IV t-PA, except in 2017. We could determine the location of large artery occlusion in 851 of 1021 patients who underwent angiographic studies before reperfusion therapy. The most common occlusion site was the middle cerebral artery (MCA) (56.1%, *n* = 477), followed by the internal carotid artery (ICA) (27.1%, *n* = 231), vertebrobasilar (VBA) artery (12.1%, *n* = 103), and others (4.7%, *n* = 40). During study period, thrombectomy devices including Solitaire, Trevo, and Penumbra system were available in Korea. Among 593 patients treated with EVT, Solitaire was most frequently selected in 433 (73%), followed by Penumbra system (*n* = 69, 11.6%), and Trevo (*n* = 69, 11.6%).

### 3.2. Secular Trends of Functional and Radiologic Outcomes

There was an increase in the number of patients who received the reperfusion therapy and the number of patients was 103 in 2012, 231 in 2013, 284 in 2014, 302 in 2015, and 306 in 2017. When we compared the baseline characteristics annually, initial diastolic blood pressure, NIHSS score at admission, and frequency of atrial fibrillation or atrial flutter decreased, while frequency of dyslipidemia or preadmission disability (mRS score of >2 before stroke) increased (Table 1). However, there were no differences in demographics or other vascular risk factors between calendar years. In time parameters, the intervals from stroke onset to arrival to emergency department (ED) increased, while those from door to initiation of IV t-PA (in 908 patients who received IV t-PA) or groin puncture (in 593 patients who received EVT) decreased over the years (all *p* < 0.05, Table 1). Especially, in 497 patients who had achieved final recanalization using EVT, the intervals from door to final recanalization [decreased from 240.1 ± 99.1 min in 2012 to 172.9 ± 62.6 min in 2017 (*p* < 0.001). During the study period, total number of stroke neurologist of study hospitals slightly increased (34 in 2012, 37 in 2013, 38 in 2014, 40 in 2015, 41 in 2017), while the number of neurointerventionalists or neurosurgeons involving EVT was similar (35 in 2012, 37 in 2013, 36 in 2014, 35 in 2015, 37 in 2017).

**Figure 2:**
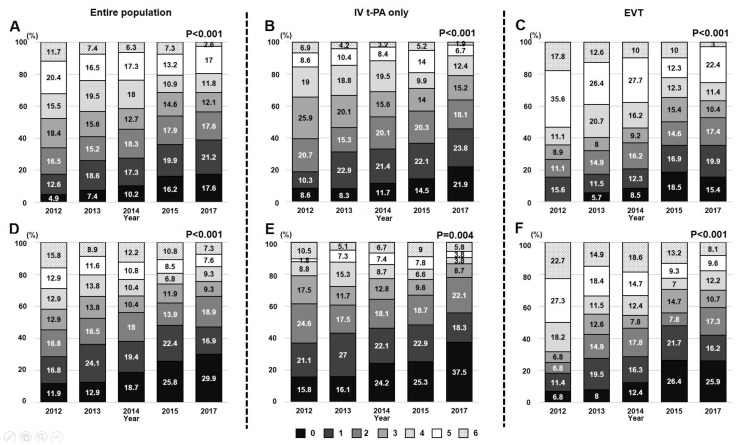
Secular trends in functional outcome at discharge (**A**–**C**) and after 90 days (**D**–**F**). Distribution of mRS scores of the entire population (A and D), those who received IV t-PA alone (**B**,**E**), and those who received EVT with/without IV t-PA (**C**,**F**).

During the study period, patients achieved favorable functional outcome (mRS score of 0–2) in 48.6% and excellent functional outcome (mRS score of 0–1) in 31.3% at discharge. Among 1203 patients with available mRS data at 90 days, 501 (41.8%) patients achieved favorable functional outcome and 703 (58.6%) patients did excellent functional outcome at 90 days. The improvement in functional outcome at discharge after reperfusion therapy was noted as calendar year increased (Figure 2). The proportion of patients who were discharged home significantly increased (from 45.6% in 2012 to 58.5% in 2017) (*p* < 0.001). Likewise, functional outcome at 90 days was also different between calendar years (*p* < 0.05). There was an increase in favorable functional outcome (mRS score of 0–2) or excellent outcome (mRS score of 0–1) at discharge or after 90 days (all *p* < 0.05) (Table 2). In addition, the mortality rate at discharge or after 90 days significantly decreased with an increase in calendar year (all *p* < 0.05). These secular trends were consistently observed regardless of treatment modalities (Figure 2).

Successful recanalization rate could be evaluated in 804 patients who had undergone follow-up angiographic studies within 24 h after IV t-PA therapy or EVT. The successful recanalization rate increased over time from 78.3% in 2012 to 85.2% in 2017 (*p* = 0.004) (Table 2). On the contrary, the development of symptomatic intracerebral hemorrhage has declined over time (*p* = 0.018).

### 3.3. Determinants of Functional Outcomes

We investigated the determinants associated with functional outcomes. The univariable analysis demonstrated that favorable outcome (mRS score of 0–2) at discharge was associated with age, male sex, diabetes, current smoking, atrial fibrillation or atrial flutter, previous stroke, preadmission disability, initial stroke severity, and intervals from stroke onset to ED or from ED to reperfusion therapy, along with the calendar year (all *p* < 0.05). Among 804 patients who had large vessel occlusion and underwent angiographic studies before and after reperfusion therapy, favorable outcome was associated with occlusion site or achievement of successful recanalization. After adjusting these significant variables (*p* < 0.05) in the univariable analysis, the independent and significant predictors for favorable functional outcome at discharge were age, diabetes, preadmission disability, initial stroke severity, intervals from door to reperfusion therapy, achievement of successful recanalization, and calendar year (Supplementary Table S1). When we investigated the independent factor for functional outcome at 90 days. the same variables were independently associated with favorable outcome at 90 days (Table 3).

Further, we performed the univariable and multivariable ordinal regression analysis to obtain a significant factor for a shift in mRS at discharge and 90 days. The independent determinants of mRS at discharge and 90 days included age, diabetes, previous stroke, preadmission disability, initial stroke severity, intervals from door to reperfusion therapy, and achievement of successful recanalization, along with the calendar year (Supplementary Table S1 and Table 3).

## 4. Discussion

We investigated whether clinical or radiologic outcomes after reperfusion therapy changed over time using nationwide, multicenter data covering real clinical practice between 2012 and 2017 in Korea. We demonstrated that there was an increase in the number of patients who received reperfusion therapy, especially EVT, and the clinical outcomes of patients who received reperfusion therapy significantly improved over the past five years. The favorable trend in functional outcomes may be partly ascribed to the increases in successful recanalization rate and decreases in the door-to-treatment intervals and hemorrhagic complications. Of note, the calendar year was a significant factor for functional outcome even after adjusting for significant determinants.

The rate of favorable outcome in this study was comparable to or even better than those of the previous randomized controlled trials. For example, in previous trials investigating the usefulness of IV t-PA, the proportions of patients with mRS score of 0–1 at 90 days were 39% in the National Institute of Neurological Disorders and Stroke study [13], 52.4% in ECASS III [12], and 39% in Safe Implementation of Thrombolysis in Stroke-Monitoring Study [17]. A previous study using the data of Get with the Guidelines-Stroke hospitals showed that in-hospital mortality and discharge-to-home rates were 8.25% and 42.7%, respectively, among patients received IV t-PA therapy [18].

In this analysis, 45.3% of patients had a mRS score of 0–1 at 90 days, and 62.4% of patients could be discharged to home among patients with IV t-PA alone. 

The meta-analysis of pooled patient data from five randomized trials after 2015 showed that 46% of patients achieved mRS score of 0–2 at 90 days [4]. In our patients who received EVT with/without IV t-PA, 50.7% of patients had an mRS score of 0–2 at 90 days. In addition, successful recanalization rate in our study population was 83%–87%, which was similar to those in recent EVT trials [19]. Although comparison of clinical outcomes between studies might be difficult because of different patient characteristics, feasibilities of procedures, types of devices, or treatment modalities between studies, our data suggested the benefits of current reperfusion therapy can be reproduced in the real clinical practice. 

Our study also demonstrated that some characteristics of patients who received the reperfusion therapy have changed. Although age, vascular risk factors other than dyslipidemia, and occlusion site did not change over time, stroke severity slightly decreased. This may be ascribed to the increase in reperfusion therapy for minor stroke and decrease in the prevalence of atrial fibrillation- or atrial flutter-related stroke [20,21]. Furthermore, time from stroke onset to ED was longer than those in previous studies because patient eligibility for EVT might be based more on imaging parameters recently, instead of the time window paradigm [22]. Increase in the intervals from stroke onset to arrival to ED over years might be partly because more patients with delayed presentation to ED might have been treated with the reperfusion therapy over the years, thanks to advances in selection of patients by multimodal neuroimaging.

In this study, we reaffirm the importance of earlier treatment and achieving successful recanalization to improve patient outcome. Reducing the time from stroke onset to treatment is beneficial to not only reduce the ischemic core but also to remove thrombus [23,24,25]. Earlier treatment would also reduce the risk of symptomatic intracranial hemorrhage. [26,27] In Korea, there have been continuing efforts on reducing the time delay for patients with stroke using the stroke code system based on the computerized physician order entry system [9,27]. Actually, our data showed that mean time from door to initiation of treatment was continuously decreasing (up to 41.1 min for IV t-PA or 113 min for groin puncture). 

Moreover, completely reopening the occluded artery is known as one of the essential components in reperfusion therapy [5]. Currently, the stentriever is the most commonly preferred device in approximately 90% of hospitals in Korea [8]. Stentriever use had some advantages such as easy handling, faster and complete reperfusion, temporal opening, and lesser bleeding complications [28,29]. There were increasing trends in successful recanalization (up to 87% in 2017) and rapid recanalization (mean intervals from door to final recanalization from 240.1 min in 2014 to 173 min in 2017) and decreasing trends in symptomatic intracranial hemorrhage, which might be related with growing use of stentriever over time in Korea. 

The calendar year was one of significant factors for favorable outcome even after adjusting for significant variables including age, initial stroke severity, comorbidities, and time from door to needle or achievement of successful recanalization. This implied that the advances in certain factors over time could also play a role for these trends. First, optimal medical treatment before and after reperfusion therapy would prevent complications or early neurologic deterioration and favorably affect the outcome. In this context, the role of the stroke team is important even in the era of EVT for acute stroke. Over the past decades, there has been an improvement in stroke care in Korea: Increase in stroke unit-based centers, multidisciplinary stroke team, guideline-based clinical practice, certification of stroke center, use of antithrombotics or statin in preventing stroke, development of networks among regional comprehensive and primary stroke centers, and provision of nationwide quality care for stroke [10,30,31,32]. Additionally, there was a possibility of advancement in the selection of eligible candidates who would benefit from reperfusion therapy. Many stroke centers in Korea use imaging parameters, such as collateral status, diffusion/perfusion mismatch, clot characteristics, or ASPECTS score, for identifying patients who are more suitable for reperfusion therapy [8,33].

There are some limitations to this study. First, the decision regarding the method of reperfusion therapy was based on discretion of the stroke neurologist or neurointerventionalist at each study center. The selection of EVT device, number of EVT passes, and use of balloon-guided catheter was not standardized. However, most stroke centers are collaborative in terms of sharing experience and protocols. Second, there were some unmeasured variables, such as socioeconomic status, educational level, and medication adherence before/after stroke, that could affect the functional outcome after stroke. 

## 5. Conclusions

Our analysis demonstrated the increase in favorable outcomes among patients who received reperfusion therapy in Korea. There were a changing patterns, such as an improvement in time parameters, increase in successful recanalization rate, and decrease in bleeding complication rate, leading to the improvement in stroke metrics. However, there is still room for additional efforts, such as rapid notification or communication, for reduction in time delay before arrival to the hospital. Our data also suggest the need for support of personnel or infrastructure to continue the optimum treatment.

## Figures and Tables

**Table 1:** Baseline characteristics of the included patients by year.

	Total	Calendar Year					*p*-Value for Trends
		2012 (*n* = 103)	2013 (*n* = 231)	2014 (*n* = 284)	2015 (*n* = 302)	2017 (*n* = 306)	
Age	68.9 ± 11.6	68.7 ± 10.3	68.8 ± 11.2	68.8 ± 11.7	70.0 ± 11.3	67.9 ± 12.6	0.941
Male sex	723 (59.0)	58 (56.3)	142 (61.5)	162 (57.0)	168 (55.6)	193 (63.1)	0.328
Hypertension	869 (70.9)	79 (76.7)	168 (72.7)	193 (68.0)	213 (70.5)	216 (70.6)	0.437
Diabetes	499 (40.7)	53 (51.5)	101 (43.7)	110 (38.7)	108 (35.8)	127 (41.5)	0.187
Hyperlipidemia	408 (33.3)	33 (32.0)	60 (26.0)	98 (34.5)	87 (28.8)	130 (42.5)	0.001
Current smoking	273 (22.3)	22 (21.4)	52 (22.5)	70 (24.6)	64 (21.2)	65 (21.2)	0.630
Coronary disease	212 (17.3)	21 (20.4)	31 (13.4)	55 (19.4)	52 (17.2)	53 (17.3)	0.903
Valvular heart disease	47 (3.8)	4 (3.9)	3 (1.3)	10 (3.5)	20 (6.6)	10 (3.3)	0.408
Mechanical valvular disease	18 (1.5)	4 (3.9)	2 (0.9)	3 (1.1)	4 (1.3)	5 (1.6)	0.714
Mitral stenosis	29 (2.4)	0 (0.0)	1 (0.4)	7 (2.5)	16 (5.3)	5 (1.6)	0.182
Atrial fibrillation or atrial flutter	592 (48.3)	55 (53.4)	112 (48.5)	144 (50.7)	159 (52.6)	122 (39.9)	0.008
Congestive heart failure	68 (5.5)	5 (4.9)	16 (6.9)	18 (6.3)	16 (5.3)	13 (4.2)	0.265
Peripheral arterial occlusive diseases	22 (1.8)	3 (2.9)	8 (3.5)	4 (1.4)	2 (2.2)	5 (1.6)	0.141
Previous stroke	246 (20.1)	26 (25.2)	42 (18.2)	60 (21.1)	55 (18.2)	63 (20.3)	0.754
Preadmission disability	50 (4.1)	8 (7.8)	10 (4.3)	16 (5.6)	8 (2.6)	8 (2.6)	0.020
Location site (*n* = 1021)							0.170
ICA	231 (22.6)	26 (34.7)	35 (19.7)	53 (24.5)	54 (21.7)	63 (20.8)	
MCA	477 (46.7)	24 (32.0)	81 (45.5)	110 (50.9)	125 (50.2)	137 (45.2)	
VBA	103 (10.1)	11 (14.7)	20 (11.2)	23 (10.6)	18 (7.2)	31 (10.2)	
Others	40 (3.9)	2 (2.7)	10 (5.6)	7 (3.2)	7 (2.8)	14 (4.6)	
No Occlusion	170 (16.7)	12 (16.0)	32 (18.0)	23 (10.6)	45 (18.1)	58 (19.1)	
Systolic blood pressure	149.9 ± 28.8	153.3 ± 33.6	152.5 ± 30.1	148.3 ± 27.6	149.2 ± 28.8	148.8 ± 27.3	0.078
Diastolic blood pressure	85 ± 16.6	87.4 ± 21.1	86.3 ± 15.9	86.3 ± 16.6	84.1 ± 16.4	82.8 ± 15.3	0.018
Last normal to ED	144.3 ± 180.4	117.6 ± 159.2	131.7 ± 146.6	139.4 ± 178.3	148.9 ± 174.5	162.8 ± 213.8	0.015
ED to CT (*n* = 1212)	18.5 ± 21.7	21.5 ± 16.1	18.4 ± 42.3	16.5 ± 12.5	17.8 ± 11.6	20.1 ± 14.1	0.450
ED to t-PA infusion (*n* = 908)	47.1 ± 23.1	56.5 ± 24.9	47.8 ± 22	47.1 ± 21.8	48.7 ± 25.5	41.1 ± 20.6	<0.001
ED to groin puncture (*n* = 593)	124.1 ± 57.7	137.2 ± 63.7	133.7 ± 66.5	131.6 ± 50.8	123.1 ± 60.3	112.8 ± 53.1	0.009
ED to final recanalization (*n* = 497)	192.3 ± 76.8	240.1 ± 99.1	206.6 ± 89.1	208.8 ± 69.2	181.8 ± 78.4	172.9 ± 62.6	<0.001
Last normal to final recanalization (*n* = 497)	389.9 ± 244.2	401.3 ± 184.8	402.1 ± 206.9	395.0 ± 249.0	395.7 ± 257.0	367.8 ± 256.6	0.127
NIHSS score at stroke onset	12 (7−17)	13 (7−18)	13 (7−17)	13 (7−18)	12 (6−17)	12 (5−16)	0.004
Treatment modality							<0.001
IV t-PA alone	633 (51.6)	58 (56.3)	144 (62.3)	154 (54.2)	172 (57.0)	105 (34.3)	
EVT alone	318 (25.9)	25 (24.3)	49 (21.2)	71 (25.0)	73 (24.2)	100 (32.7)	
EVT + IV t-PA	275 (22.4)	20 (19.4)	38 (16.5)	59 (20.8)	57 (18.9)	101 (33.0)	
Use of stentriever *	499 (48.1)	31 (68.9)	60 (69.0)	107 (82.3)	115 (88.5)	186 (92.5)	<0.001

Values are presented as *n* (%), unless otherwise indicated. ICA, internal carotid artery; MCA, middle cerebral artery; VBA, vertebrobasilar artery; ED, emergency department; NIHSS, National Institutes of Health Stroke Scale; IV, intravenous; EVT, endovascular; t-PA, tissue plasminogen activator. * Among 499 patients who received EVT with/without IV t-PA.

**Table 2:** Clinical and radiologic outcome.

	Total	Calendar Year					*p*-Value for Trends
		2012	2013	2014	2015	2017	
mRS score of 0–1 at discharge	384 (31.3)	18 (17.5)	60 (26.0)	78 (27.5)	109 (36.1)	119 (38.9)	<0.001
mRS score of 0–2 at discharge	596 (48.6)	35 (34.0)	95 (41.1)	130 (45.8)	163 (54.0)	173 (56.5)	<0.001
Discharge route							<0.001
Transfer to rehabilitation	168 (13.7)	18 (17.5)	25 (10.8)	42 (14.8)	27 (8.9)	56 (18.3)	
Home	662 (54.0)	47 (45.6)	114 (49.4)	140 (49.3)	182 (60.3)	179 (58.5)	
Transfer to other hospitals or departments	318 (25.9)	26 (25.2)	76 (32.9)	82 (28.9)	71 (23.5)	63 (20.6)	
Death	78 (6.4)	12 (11.7)	16 (6.9)	20 (7.0)	22 (7.3)	8 (2.6)	
mRS score of 0–1 at 90 days	501 (41.8)	29 (28.7)	83 (37.1)	106 (38.1)	142 (48.1)	141 (46.8)	<0.001
mRS score of 0–2 at 90 days	703 (58.6)	46 (45.5)	120 (53.6)	156 (56.1)	183 (62.0)	198 (65.8)	<0.001
Successful recanalization immediately after EVT	478 (80.6)	37 (82.2)	66 (75.9)	99 (76.2)	101 (77.7)	175 (87.1)	0.02
Successful recanalization within 24 h	628 (78.1)	47 (78.3)	95 (71.4)	135 (75.8)	143 (75.7)	208 (85.2)	0.004
Symptomatic intracranial hemorrhage	46 (3.8)	4 (3.9)	13 (5.6)	12 (4.2)	13 (4.3)	4 (1.3)	0.018

Values are presented as *n* (%), unless otherwise indicated. mRS, modified Rankin score; EVT, endovascular treatment.

**Table 3:** Independent determinants for functional outcome at 90 days.

	Ordinal Logistic Regression (Increase in mRS)				Binary Logistic Regression (mRS ≤ 2 vs. mRS > 2)			
	OR (95% CI)	*p*-Value *	OR (95% CI)	*p*-Value ^†^	OR (95% CI)	*p*-Value *	OR (95% CI)	*p*-Value ^†^
Calendar year								
2012	1		1		1		1	
2013	0.882 (0.579–1.343)	0.559	0.686 (0.391–1.203)	0.188	1.209 (0.701–2.088)	0.495	1.776 (0.813–3.882)	0.15
2014	0.796 (0.529–1.198)	0.274	0.579 (0.339–0.991)	0.046	1.464 (0.857–2.501)	0.163	2.650 (1.241–5.661)	0.012
2015	0.626 (0.417–0.942)	0.025	0.525 (0.306–0.900)	0.019	1.706 (1.000–2.909)	0.05	2.014 (0.950–4.273)	0.068
2017	0.507 (0.337–0.762)	0.001	0.467 (0.276–0.791)	0.005	2.131 (1.246–3.645)	0.006	2.395 (1.142–5.023)	0.021
Age	1.03 (1.019–1.04)	<0.001	1.027 (1.014–1.039)	<0.001	0.966 (0.953–0.980)	<0.001	0.968 (0.951–0.985)	<0.001
Male sex	0.936 (0.746–1.173)	0.565	0.85 (0.642–1.125)	0.255	1.083 (0.802–1.461)	0.604	1.262 (0.857–1.859)	0.239
Diabetes	2.421 (1.955–2.997)	<0.001	2.791 (2.139–3.643)	<0.001	0.382 (0.290–0.503)	<0.001	0.329 (0.230–0.469)	<0.001
Current smoking	1.037 (0.785–1.369)	0.799	0.912 (0.634–1.313)	0.621	0.976 (0.673–1.417)	0.9	1.112 (0.672–1.842)	0.679
Atrial fibrillation or atrial flutter	0.721 (0.579–0.897)	0.003	0.8 (0.613–1.046)	0.103	1.468 (1.100–1.959)	0.009	1.216 (0.843–1.753)	0.296
Congestive heart failure	1.294 (0.823–2.035)	0.265	1.333 (0.801–2.219)	0.269				
Previous stroke	1.359 (1.05–1.759)	0.02	1.418 (1.05–1.914)	0.023	0.742 (0.530–1.039)	0.082	0.793 (0.526–1.195)	0.267
Preadmission disability	3.888 (2.268–6.673)	<0.001	2.855 (1.565–5.202)	0.001	0.094 (0.035–0.251)	<0.001	0.178 (0.062–0.510)	0.001
Last normal to ED	1.001 (1.000–1.001)	0.08	1.001 (1.000–1.001)	0.095	0.999 (0.999–1.000)	0.104	0.999 (0.999–1.000)	0.126
ED to treatment	1.003 (1.001–1.005)	0.002	1.003 (1.001–1.005)	0.002	0.996 (0.994–0.999)	0.002	0.995 (0.992–0.998)	0.001
NIHSS at stroke onset	1.119 (1.099–1.139)	<0.001	1.126 (1.100–1.152)	<0.001	0.878 (0.857–0.900)	<0.001	0.860 (0.832–0.890)	<0.001
Location site								
ICA			1				1	
MCA			0.871 (0.647–1.174)	0.365			0.834 (0.554–1.257)	0.386
VBA			1.215 (0.787–1.878)	0.379			0.723 (0.391–1.338)	0.302
Others			1.212 (0.608–2.414)	0.584			0.544 (0.211–1.403)	0.208
Successful recanalization within 24 h			0.221 (0.159–0.307)	<0.001			6.477 (4.056–10.345)	<0.001

ED, emergency department; NIHSS, National Institutes of Health Stroke Scale; ICA, internal carotid artery; MCA, middle cerebral artery; VBA, vertebrobasilar artery. * Adjusted for significant variables in the univariable analysis among the entire study population (*n* = 1199). ^†^ Adjusted for significant variables in the univariable analysis among patients who had arterial occlusion at initial angiographic studies and could determine whether a successful recanalization was achieved at follow-up angiographic studies (*n* = 783).

## Supplementary Materials

The following are available online at https://www.mdpi.com/2077-0383/9/3/717/s1, Table S1: Independent determinants for functional outcome at discharge.

## References

[1] Wang H., Naghavi M., Allen C., Barber R.M., Bhutta Z.A. (2016). Global, regional, and national life expectancy, all-cause mortality, and cause-specific mortality for 249 causes of death, 1980–2015: A systematic analysis for the global burden of disease study 2015. Lancet (Lond. Engl.).

[2] Kassebaum N.J., Arora M., Barber R.M., Bhutta Z.A., Brown J., Carter A., Casey D.C., Charlson F.J., Coates M.M., Coggeshall M. (2016). Global, regional, and national disability-adjusted life-years (dalys) for 315 diseases and injuries and healthy life expectancy (hale), 1990–2015: A systematic analysis for the global burden of disease study 2015. Lancet.

[3] Donnan G.A., Fisher M., Macleod M., Davis S.M. (2008). Stroke. Lancet (Lond. Engl.).

[4] Goyal M., Menon B.K., van Zwam W.H., Dippel D.W., Mitchell P.J., Demchuk A.M., Davalos A., Majoie C.B., van der Lugt A., de Miquel M.A. (2016). Endovascular thrombectomy after large-vessel ischaemic stroke: A meta-analysis of individual patient data from five randomised trials. Lancet (Lond. Engl.).

[5] Rha J.H., Saver J.L. (2007). The impact of recanalization on ischemic stroke outcome: A meta-analysis. Stroke.

[6] Fargen K.M., Meyers P.M., Khatri P., Mocco J. (2013). Improvements in recanalization with modern stroke therapy: A review of prospective ischemic stroke trials during the last two decades. J. Neurointerv. Surg..

[7] Jansen I.G.H., Mulder M., Goldhoorn R.B., investigators M.C.R. (2018). Endovascular treatment for acute ischaemic stroke in routine clinical practice: Prospective, observational cohort study (mr clean registry). BMJ.

[8] Seo K.-D., Suh S.H. (2018). Endovascular treatment in acute ischemic stroke: A nationwide survey in korea. Neurointervention.

[9] Heo J.H., Kim Y.D., Nam H.S., Hong K.S., Ahn S.H., Cho H.J., Choi H.Y., Han S.W., Cha M.J., Hong J.M. (2010). A computerized in-hospital alert system for thrombolysis in acute stroke. Stroke.

[10] Kim J.Y., Kang K., Kang J., Koo J., Kim D.H., Kim B.J., Kim W.J., Kim E.G., Kim J.G., Kim J.M. (2019). Executive summary of stroke statistics in korea 2018: A report from the epidemiology research council of the korean stroke society. J. Stroke.

[11] Goldstein L.B., Samsa G.P., Matchar D.B., Horner R.D. (2004). Charlson index comorbidity adjustment for ischemic stroke outcome studies. Stroke.

[12] Hacke W., Kaste M., Bluhmki E., Brozman M., Dávalos A., Guidetti D., Larrue V., Lees K.R., Medeghri Z., Machnig T. (2008). Thrombolysis with alteplase 3 to 4.5 h after acute ischemic stroke. N. Engl. J. Med..

[13] Tissue Plasminogen Activator for Acute Ischemic Stroke (1995). The national institute of neurological disorders and stroke rt-pa stroke study group. N. Engl. J. Med..

[14] Jauch E.C., Saver J.L., Adams H.P., Bruno A., Connors J.J., Demaerschalk B.M., Khatri P., McMullan P.W., Qureshi A.I., Rosenfield K. (2013). Guidelines for the early management of patients with acute ischemic stroke: A guideline for healthcare professionals from the american heart association/american stroke association. Stroke.

[15] Kernan W.N., Ovbiagele B., Black H.R., Bravata D.M., Chimowitz M.I., Ezekowitz M.D., Fang M.C., Fisher M., Furie K.L., Heck D.V. (2014). Guidelines for the prevention of stroke in patients with stroke and transient ischemic attack: A guideline for healthcare professionals from the american heart association/american stroke association. Stroke.

[16] Higashida R.T., Furlan A.J. (2003). Trial design and reporting standards for intra-arterial cerebral thrombolysis for acute ischemic stroke. Stroke.

[17] Wahlgren N., Ahmed N., Davalos A., Ford G.A., Grond M., Hacke W., Hennerici M.G., Kaste M., Kuelkens S., Larrue V. (2007). Thrombolysis with alteplase for acute ischaemic stroke in the safe implementation of thrombolysis in stroke-monitoring study (sits-most): An observational study. Lancet.

[18] Fonarow G.C., Zhao X., Smith E.E., Saver J.L., Reeves M.J., Bhatt D.L., Xian Y., Hernandez A.F., Peterson E.D., Schwamm L.H. (2014). Door-to-needle times for tissue plasminogen activator administration and clinical outcomes in acute ischemic stroke before and after a quality improvement initiative. JAMA.

[19] Hong K.S., Ko S.B., Lee J.S., Yu K.H., Rha J.H. (2015). Endovascular recanalization therapy in acute ischemic stroke: Updated meta-analysis of randomized controlled trials. J. Stroke.

[20] Baek J.H., Kim B.M. (2019). Angiographical identification of intracranial, atherosclerosis-related, large vessel occlusion in endovascular treatment. Front. Neurol..

[21] Yoo J., Sohn S.I., Kim J., Ahn S.H., Lee K., Baek J.H., Kim K., Hong J.H., Koo J., Kim Y.D. (2018). Delayed intravenous thrombolysis in patients with minor stroke. Cerebrovasc. Dis..

[22] Kim J.T., Cho B.H., Choi K.H., Park M.S., Kim B.J., Park J.M., Kang K., Lee S.J., Kim J.G., Cha J.K. (2019). Magnetic resonance imaging versus computed tomography angiography based selection for endovascular therapy in patients with acute ischemic stroke. Stroke.

[23] Meretoja A., Keshtkaran M., Saver J.L., Tatlisumak T., Parsons M.W., Kaste M., Davis S.M., Donnan G.A., Churilov L. (2014). Stroke thrombolysis: Save a minute, save a day. Stroke.

[24] Kim Y.D., Nam H.S., Kim S.H., Kim E.Y., Song D., Kwon I., Yang S.H., Lee K., Yoo J., Lee H.S. (2015). Time-dependent thrombus resolution after tissue-type plasminogen activator in patients with stroke and mice. Stroke.

[25] Bourcier R., Goyal M., Liebeskind D.S., Muir K.W., Desal H., Siddiqui A.H., Dippel D.W.J., Majoie C.B., van Zwam W.H., Jovin T.G. (2019). Association of time from stroke onset to groin puncture with quality of reperfusion after mechanical thrombectomy: A meta-analysis of individual patient data from 7 randomized clinical trials. JAMA Neurol..

[26] Fonarow G.C., Smith E.E., Saver J.L., Reeves M.J., Bhatt D.L., Grau-Sepulveda M.V., Olson D.M., Hernandez A.F., Peterson E.D., Schwamm L.H. (2011). Timeliness of tissue-type plasminogen activator therapy in acute ischemic stroke: Patient characteristics, hospital factors, and outcomes associated with door-to-needle times within 60 min. Circulation.

[27] Jeon S.B., Ryoo S.M., Lee D.H., Kwon S.U., Jang S., Lee E.J., Lee S.H., Han J.H., Yoon M.J., Jeong S. (2017). Multidisciplinary approach to decrease in-hospital delay for stroke thrombolysis. J. Stroke.

[28] Song D., Heo J.H., Kim D.I., Kim D.J., Kim B.M., Lee K., Yoo J., Lee H.S., Nam H.S., Kim Y.D. (2015). Impact of temporary opening using a stent retriever on clinical outcome in acute ischemic stroke. PLoS ONE.

[29] Touma L., Filion K.B., Sterling L.H., Atallah R., Windle S.B., Eisenberg M.J. (2016). Stent retrievers for the treatment of acute ischemic stroke: A systematic review and meta-analysis of randomized clinical trials. JAMA Neurol..

[30] Choi H.Y., Cha M.J., Nam H.S., Kim Y.D., Hong K.S., Heo J.H., Korean Stroke Unit Study C. (2012). Stroke units and stroke care services in korea. Int. J. Stroke.

[31] Kim Y.D., Jung Y.H., Saposnik G. (2016). Traditional risk factors for stroke in east asia. J. Stroke.

[32] Kim J., Hwang Y.H., Kim J.T., Choi N.C., Kang S.Y., Cha J.K., Ha Y.S., Shin D.I., Kim S., Lim B.H. (2014). Establishment of government-initiated comprehensive stroke centers for acute ischemic stroke management in south korea. Stroke.

[33] Yoo J., Baek J.H., Park H., Song D., Kim K., Hwang I.G., Kim Y.D., Kim S.H., Lee H.S., Ahn S.H. (2018). Thrombus volume as a predictor of nonrecanalization after intravenous thrombolysis in acute stroke. Stroke.

