# Supplementary material for: Improving the Clinical Outcome in Stroke Patients Receiving Thrombolytic or Endovascular Treatment in Korea: from the SECRET Study
Source: J Clin Med. 2020 Mar 6;9(3):717. doi: 10.3390/jcm9030717 (PMC7141338; doi:10.3390/jcm9030717)
Supplement: Supplementary file 1 [file jcm-09-00717-s001.pdf]

Supplementary Material
Improving the clinical outcome in stroke patients receiving thrombolytic or endovascular treatment in Korea: from the SECRET study

Supplementary Table S1. Independent determinants for functional outcome at discharge

	Ordinal logistic regression (increase in mRS)				Binary logistic regression (mRS ≤2 vs. mRS >2)			
	OR (95% CI)	P*	OR (95% CI)	P†	OR (95% CI)	P*	OR (95% CI)	P†
Calendar year								
2012	1		1		1		1	
2013	0.83 (0.548–1.256)	0.377	0.7 (0.401–1.224)	0.211	1.214 (0.708–2.083)	0.481	1.440 (0.650–3.189)	0.369
2014	0.727 (0.486–1.088)	0.122	0.557 (0.327–0.95)	0.032	1.564 (0.923–2.651)	0.096	2.190 (1.019–4.708)	0.045
2015	0.564 (0.377–0.843)	0.005	0.492 (0.288–0.84)	0.009	2.075 (1.230–3.502)	0.006	2.255 (1.054–4.824)	0.036
2017	0.481 (0.322–0.72)	<0.001	0.5 (0.297–0.842)	0.009	2.294 (1.358–3.874)	0.002	2.259 (1.076–4.744)	0.031
Age	1.026 (1.017–1.036)	<0.001	1.024 (1.012–1.037)	<0.001	0.973 (0.961–0.985)	<0.001	0.973 (0.958–0.989)	0.001
Male sex	0.957 (0.766–1.195)	0.698	0.959 (0.727–1.265)	0.768	1.031 (0.776–1.371)	0.832	1.121 (0.770–1.633)	0.551
Diabetes	2.352 (1.906–2.903)	<0.001	2.811 (2.157–3.665)	<0001	0.462 (0.355–0.600)	<0.001	0.387 (0.274–0.548)	<0.001
Current smoking	1.139 (0.867–1.497)	0.349	1.067 (0.744–1.530)	0.724	0.998 (0.706–1.410)	0.991	1.067 (0.657–1.730)	0.794
Atrial fibrillation or atrial flutter	0.755 (0.609–0.935)	0.01	0.8 (0.614–1.043)	0.099	1.383 (1.052–1.819)	0.02	1.169 (0.822–1.664)	0.384
Congestive heart failure	1.014 (0.652–1.579)	0.95	1.032 (0.627–1.699)	0.9				
Previous stroke	1.303 (1.009–1.683)	0.042	1.361 (1.009–1.835)	0.043	0.815 (0.586–1.134)	0.225	0.885 (0.590–1.327)	0.554
Preadmission disability	3.658 (2.151–6.215)	<0.001	3.148 (1.719–5.760)	<0.001	0.058 (0.014–0.247)	<0.001	0.053 (0.007–0.406)	0.005

Last normal to ED	1.001 (1.000–1.001)	0.067	1 (1.000–1.001)	0.13	0.999 (0.999–1.000)	0.146	0.999 (0.999–1.000)	0.225
ED to treatment	1.002 (1.001–1.004)	0.01	1.003 (1.000–1.005)	0.019	0.998 (0.995–1.000)	0.047	0.997 (0.994–1.000)	0.060
NIHSS at stroke onset	1.124 (1.104–1.144)	<0.001	1.124 (1.099–1.150)	<0.001	0.888 (0.868–0.909)	<0.001	0.880 (0.852–0.909)	<0.001
Location site								
ICA			1				1	
MCA			1.018 (0.757–1.368)	0.907			0.706 (0.474–1.053)	0.088
VBA			1.365 (0.887–2.102)	0.157			0.395 (00212–0.735)	0.003
Others			1.251 (0.636–2.46)	0.516			0.718 (0.288–1.788)	0.477
Successful recanalization within 24 h			0.186 (0.134–0.259)	<0.001			6.942 (4.195–11.487)	<0.001

ED, emergency department; NIHSS, National Institutes of Health Stroke Scale; ICA, internal carotid artery; MCA, middle cerebral artery; VBA, vertebrobasilar artery.

*Adjusted for significant variables in the univariable analysis among the entire study population (n=1226)

†Adjusted for significant variables in the univariable analysis among patients who had arterial occlusion at initial angiographic studies and could determine whether a successful recanalization was achieved at follow-up angiographic studies (n=792)
